# The point mutation UCH-L1 C152A protects primary neurons against cyclopentenone prostaglandin-induced cytotoxicity: implications for post-ischemic neuronal injury

**DOI:** 10.1038/cddis.2015.323

**Published:** 2015-11-05

**Authors:** H Liu, W Li, M E Rose, R W Hickey, J Chen, G T Uechi, M Balasubramani, B W Day, K V Patel, S H Graham

**Affiliations:** 1 Geriatric Research Educational and Clinical Center, VA Pittsburgh Healthcare System, Pittsburgh, PA, USA; 2 Department of Neurology, University of Pittsburgh School of Medicine, Pittsburgh, PA, USA; 3 Department of Pediatrics, University of Pittsburgh School of Medicine, Pittsburgh, PA, USA; 4 Genomics and Proteomics Core Laboratories, University of Pittsburgh, Pittsburgh, PA, USA; 5 Department of Pharmaceutical Sciences, University of Pittsburgh School of Pharmacy, Pittsburgh, PA, USA

## Abstract

Cyclopentenone prostaglandins (CyPGs), such as 15-deoxy-Δ^12,14^-prostaglandin J_2_ (15dPGJ2), are reactive prostaglandin metabolites exerting a variety of biological effects. CyPGs are produced in ischemic brain and disrupt the ubiquitin-proteasome system (UPS). Ubiquitin-C-terminal hydrolase L1 (UCH-L1) is a brain-specific deubiquitinating enzyme that has been linked to neurodegenerative diseases. Using tandem mass spectrometry (MS) analyses, we found that the C152 site of UCH-L1 is adducted by CyPGs. Mutation of C152 to alanine (C152A) inhibited CyPG modification and conserved recombinant UCH-L1 protein hydrolase activity after 15dPGJ2 treatment. A knock-in (KI) mouse expressing the UCH-L1 C152A mutation was constructed with the bacterial artificial chromosome (BAC) technique. Brain expression and distribution of UCH-L1 in the KI mouse was similar to that of wild type (WT) as determined by western blotting. Primary cortical neurons derived from KI mice were resistant to 15dPGJ2 cytotoxicity compared with neurons from WT mice as detected by the WST-1 cell viability assay and caspase-3 and poly ADP ribose polymerase (PARP) cleavage. This protective effect was accompanied with significantly less ubiquitinated protein accumulation and aggregation as well as less UCH-L1 aggregation in C152A KI primary neurons after 15dPGJ2 treatment. Additionally, 15dPGJ2-induced axonal injury was also significantly attenuated in KI neurons as compared with WT. Taken together, these studies indicate that UCH-L1 function is important in hypoxic neuronal death, and the C152 site of UCH-L1 has a significant role in neuronal survival after hypoxic/ischemic injury.

Ubiquitin C-terminal hydrolase L1 is a multifunctional protein that is highly expressed in neurons throughout brain.^1^ UCH-L1 closely interacts with proteins of the neuronal cytoskeleton and may have an important role in axonal transport and maintaining axonal integrity.^2, 3^ UCH-L1 regulates synaptic function and long-term potentiation (LTP) under normal and pathological conditions and may be involved in memory function.^4^ Mutations and altered function of UCH-L1 have been associated with neurological diseases including Parkinson's (PD) and Alzheimer's (AD) diseases and early onset neurodegeneration involving white matter.^2, 3, 4, 5, 6, 7^ However, the role of UCH-L1 function in cerebral ischemic injury and recovery has not been thoroughly investigated.

Cyclopentenone prostaglandins (CyPGs) are the reactive metabolites of prostaglandins containing a carbonyl moiety that may covalently modify cysteine in a variety of proteins.^8, 9, 10^ CyPG concentration is dramatically increased in ischemic brain.^11^ CyPGs such as 15dPGJ2 disrupt the ubiquitin-proteasome system (UPS), resulting in accumulation and aggregation of ubiquitinated (Ub) proteins and neuronal cell death.^12, 13^

UCH-L1 is a target of CyPG modification.^13, 14, 15^ In the current study, mass spectrometry (MS)/MS was used to determine that cysteine152 is the binding site of the CyPG 15dPGJ2 to UCH-L1. We then constructed a knock-in (KI) mouse using the bacterial artificial chromosome (BAC) technique with a cysteine to alanine mutation at this 15dPGJ2 binding site on UCH-L1. Primary neurons derived from KI and wild-type (WT) mice were used to determine the effect of CyPG binding to UCH-L1 on cell death and disruption of the UPS. These studies address a potential role for modification of UCH-L1 by CyPGs and other reactive lipid species in stroke and neurodegenerative diseases.

## Results

### C152 of UCH-L1 is the critical site for 15dPGJ2 modification

Our and others' previous work has demonstrated that 15dPGJ2 can modify UCH-L1 on cysteine residues through Michael addition and therefore profoundly alter the protein's folding and functions.^14, 15^ UCH-L1 has six cysteine residues with C90 considered as the core for protein hydrolase activity. To explore which cysteine residues in UCH-L1 are the primary targets for 15dPGJ2 adduction, Flag-tagged UCH-L1 was overexpressed in the rat primary neurons by lentivirus (LV)-Flag UCHL-WT infection and cells were then incubated with 15dPGJ2 (20 *μ*M) for 24 h before harvest. Flag-tagged UCH-L1 was immunoprecipitated with an anti-Flag antibody and the resulting precipitants were separated by PAGE, digested with trypsin, and subjected to nanoLC-MS and -MS/MS analyses.

**Figure 1:**
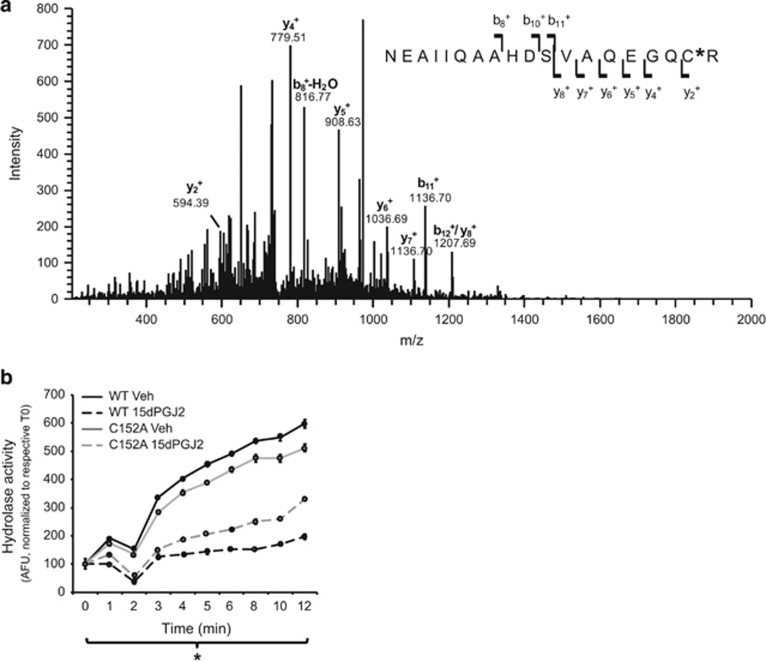
Adduct formation by 15dPGJ2 with cysteine152 is associated with decreased UCH-L1 hydrolase activity. (**a**) MS/MS spectrum of a tryptic fragment derived from Flag-tagged UCH-L1 expressed in primary neuronal cells following incubation with 15dPGJ2. Schematic representation of the amino-acid sequence, fragment ions, and the corresponding *m/z* values for the cysteine-modified tryptic peptide NEAIIQAAHDSVAQEGQC*R. 15dPGJ2 adduction (*) is shown to occur at C152. The spectrum was from an average of six tandem mass spectra from the doubly charged peptide ion at *m/z* 748.36 observed as eluted from C18 nanoLC separation with a retention range of 56.87–57.07 min. (**b**) Hydrolase activity in recombinant wild-type (WT) and mutant UCH-L1 C152A (C152A) proteins after incubation with 12.5 *μ*M 15dPGJ2 for 2 h measured at 0–12 min post substrate addition. Data are in arbitrary fluorescence units (AFUs) normalized to their respective time 0 and are expressed as means±S.E. *n*=2 per group. *P<0.05 between recombinant UCH-L1 C152A and WT 15dPGJ2-treated groups using repeated measures ANOVA with Bonferroni *post hoc* testing

One of the peptides from the digest with a retention time range of 56.87–57.07 min was observed in the MS experiment as a doubly charged ion at *m/z*=748.36. This ion yielded the MS/MS spectrum (from an average of six tandem mass spectra) shown in Figure 1a. The fragmentation pattern was consistent with the cysteine-modified tryptic peptide NEAIIQAAHDSVAQEGQC*R, indicating that 15dPGJ2 adduction (*) occurred at C152.

As a deubiquitinating enzyme, UCH-L1 hydrolase activity cleaves ubiquitin from a poly-ubiquitinated (Ub) protein. In our previous report, 15dPGJ2 modification of UCH-L1 abolished the protein's hydrolase activity.^14^ To examine whether the C152A mutation preserves hydrolase activity after incubation with 15dPGJ2, an *in vitro* hydrolase activity assay was performed using recombinant UCH-L1 WT and mutant C152A proteins with ubiquitin-AMC, a substrate that fluoresces when hydrolyzed by UCH-L1. Recombinant proteins were incubated with 12.5 *μ*M 15dPGJ2 and hydrolase activity was measured at 0–12 min after the addition of ubiquitin-AMC. As shown in Figure 1b, treatment with 15dPGJ2 decreased hydrolase activity in both WT and C152A recombinant UCH-L1 protein. However, this reduction was attenuated in mutant UCH-L1 C152A protein compared with WT, suggesting that the mutation preserves some protein function after insult by 15dPGJ2.

### Overexpression of UCH-L1 C152A protects rat primary neurons against 15dPGJ2-induced cell toxicity

**Figure 2:**
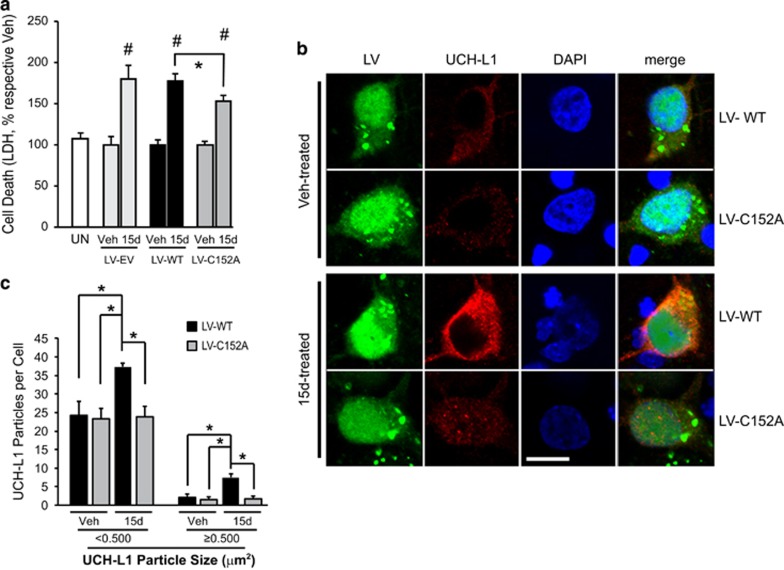
Overexpression of UCH-L1 C152A in rat primary neurons protects cells against 15dPGJ2-induced protein aggregation and cell death. Rat primary neurons were infected with flag-tagged lentivirus (LV)-UCH-L1 wild-type (WT) or LV-UCH-L1 C152A (C152A) at DIV2, then treated with 5 *μ*M 15dPGJ2 (15d) or vehicle (Veh) for 48 h at DIV10. (**a**) Cell death as measured by LDH release, normalized to respective vehicle control. *N*=6–12 per group. *P>0.05; ^#^*P*<0.01 *versus* UN; Student's *t*-test. (**b**) Representative photos of LV-infected rat primary neurons after anti-flag immunocytochemistry (red, UCH-L1). Green is EGFP (indicating lentiviral infection) and blue is DAPI nuclear stain. Bar=10 *μ*m. Photos taken with an Olympus confocal microscope at 240 × . (**c**) UCH-L1 particle counts per cell: UCH-L1 particle sizes were measured and counted using NIH ImageJ software (National Institutes of Health). *n*=23–25 per group. *P<0.05 using repeated measures ANOVA with Bonferroni *post hoc* testing. (**a** and **c**) Data are means±S.E.

To test the effect of the UCH-L1 C152A mutation on 15dPGJ2-induced primary neuron injury, a series of lentiviral vectors was constructed to infect and overexpress WT UCH-L1 or mutant UCH-L1 C152A in primary neuronal cells. Cells were infected with flag-tagged LV-UCH-L1 WT or LV-UCH-L1 C152A (C152A) at 2 *days in vitro* (DIV2), and then treated with 5 *μ*M 15dPGJ2 or vehicle for 48 h. Cell death was measured by lactate dehydrogenase (LDH) assay. As shown in Figure 2a, 15dPGJ2 significantly induced primary neuronal cell death and this toxic effect was attenuated by the overexpression of the UCH-L1 C152A mutation. To analyze the distribution of overexpressed UCH-L1, a separate cohort of cells was prepared identically and fixed with paraformaldehyde (PFA) followed by immunocytochemistry with anti-flag antibody. UCH-L1 particle sizes were measured using ImageJ software (National Institutes of Health) and were divided into two categories: large particles measuring over 0.5 *μ*M^2^ and small particles measuring less than 0.5 *μ*M^2^. The numbers of large and small particles per cell were counted in both UCH-L1 WT and C152A overexpressing primary neurons after 15dPGJ2 or vehicle treatment. 15dPGJ2-induced UCH-L1 protein aggregation was observed in primary neurons overexpressing WT UCH-L1; this effect was not observed in cells overexpressing the UCH-L1 C152A mutation (Figures 2b and c).

### Construction of the UCH-L1 C152A KI mouse

**Figure 3:**
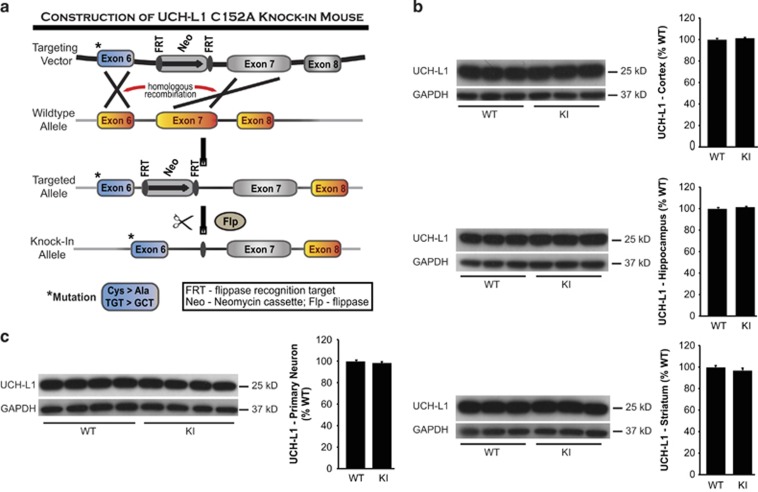
Generation of the UCH-L1 C152A knock-in (KI) mouse. (**a**) Schematic representation of homologous recombination of DNA fragments producing a point mutation in UCH-L1 converting the 152 cysteine to alanine. (**b**) UCH-L1 protein expression in UCH-L1 C152A KI and wild-type (WT) mouse brain cortex, hippocampus, and striatum. Brain regions (*n*=3 per group) were lysed and immunoblotted using anti-UCH-L1 and anti-GAPDH antibodies. *Left:* immunoblots; *right:* Graphical densitometric immunoblot analysis. (**c**) UCH-L1 protein expression in mouse UCH-L1 WT and KI primary neurons produced from UCH-L1 C152A KI and WT mice (*n*=4 per group). *Left:* immunoblots; *right:* Graphical densitometric immunoblot analysis. (**b** and **c**) Data are means±S.E. and normalized to their respective WT groups. GAPDH was used as a loading control

UCH-L1 is expressed solely in the central nervous system and the reproductive system, making primary neurons an ideal *in vitro* model in which to investigate the functions of UCH-L1. However, because the protein level of endogenous UCH-L1 is very high in primary neurons and because the lentiviral infection rate was limited to 40–50% in primary neurons, a KI mouse expressing the UCH-L1 C152A mutation was developed to overcome these obstacles. In collaboration with the University of Michigan Transgenic Core, a targeting vector was constructed and UCH-L1 C152A heterozygous KI male mice were produced on a C57Bl6 background (Figure 3a). These males were backbred to C57Bl6 female mice, and the resulting heterozygous offspring were crossbred to produce homozygous mutant UCH-L1 C152A and WT UCH-L1 lines. No significant differences in growth rate, reproductive abilities, or gross behavior were observed in the KI mice, as compared with WT. The distribution pattern and expression levels of UCH-L1 were also similar between WT and KI mice (Figure 3b). In addition, UCH-L1 expression was the same in primary neurons obtained from UCH-L1 C152A KI mice compared with neurons from WT mice (Figure 3c).

### Primary neurons from UCH-L1 C152A KI mice are resistant to 15dPGJ2-induced cell death

**Figure 4:**
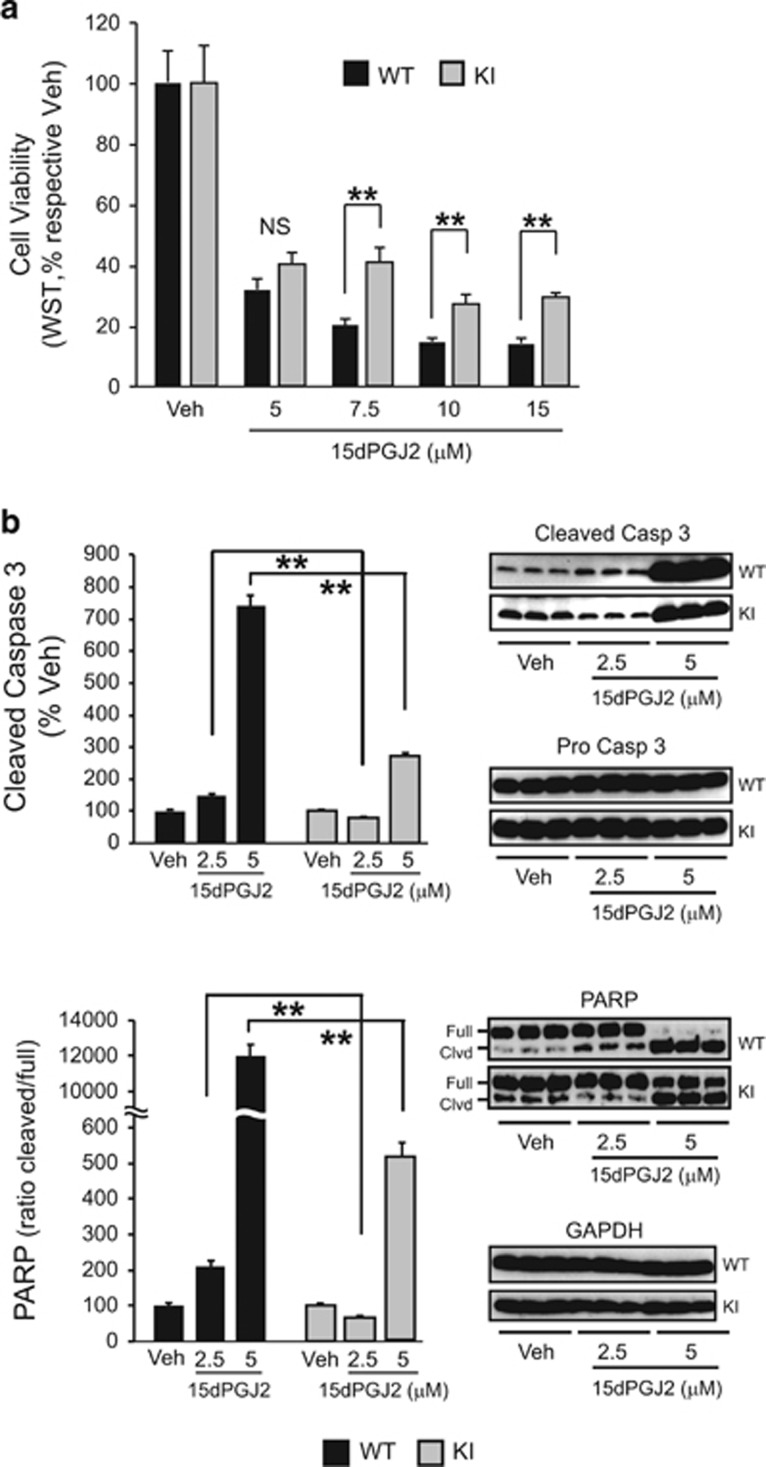
The UCH-L1 C152A mutation confers protection against 15dPGJ2-induced apoptotic cell death. (**a** and **b**) UCH-L1 C152A knock-in (KI) and wild-type (WT) primary neurons were treated with 15dPGJ2 or vehicle (DMSO, Veh) for 24 h. (**a**) Cell viability (WST-1 assay) after treatment with 5–15 *μ*M 15dPGJ2. *N*=6 per group. (**b**) Immunoblot detection of cleaved caspase 3 (Casp 3), pro Casp 3 (upper group), and PARP (full length: full, cleaved: clvd, lower group) using anti-caspase 3 and anti-PARP antibodies, respectively, after treatment with 2.5 or 5 *μ*M 15dPGJ2. GAPDH was used as a loading control. Left: Graphical densitometric analysis of immunoblots (right). *N*=3 per group. All: Data are means±S.E. and are normalized to their respective vehicle controls. **P<0.01 *versus* WT; NS, not significant. Black bar: WT; gray bar: UCH-L1 C152A

To assess the effect of the UCH-L1 C152A mutation on cell viability, primary neurons from C152A KI mice were cultured *in vitro* for 9 days before treatment with 15dPGJ2 or vehicle at 5–15 *μ*M for 24 h. Compared with primary neurons from WT mice, primary neurons produced from UCH-L1 C152A mutant mice were more resistant to 15dPGJ2 cytotoxicity as indicated by significantly higher cell viability after treatment (Figure 4a). Consistent with this finding, protective effects of the UCH-L1 C152A mutation were further confirmed by immunoblotting. When primary neurons were treated with 15dPGJ2 at 2.5 or 5 *μ*M for 24 h, increases in cleaved caspase 3 and poly ADP ribose polymerase (PARP) were observed, suggesting the activation of the apoptosis signal transduction pathway. The activation of caspase 3 and PARP was attenuated in cells produced from UCH-L1 C152A KI mice as compared with WT, indicating that 15dPGJ2-induced apoptosis was significantly inhibited in KI cells (Figure 4b).

### Primary neurons from UCH-L1 C152A KI mice are resistant to 15dPGJ2-induced protein aggregation

**Figure 5:**
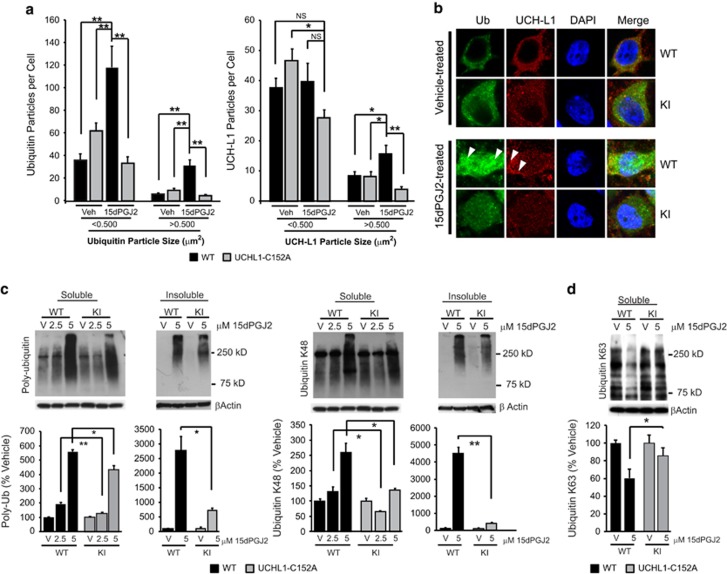
The UCH-L1 C152A mutation attenuates 15dPGJ2-induced protein aggregation in primary neurons. (**a** and **b**) Wild type (WT) and UCH-L1 C152A knock-in (KI) primary neurons were treated with 2.5 *μ*M 15dPGJ2 or vehicle (DMSO, Veh) for 24 h then immunostained with anti-ubiquitin (green) and anti-UCH-L1 (red) antibodies. (**a**) Ubiquitin (left) and UCH-L1 (right) particles were counted using ImageJ software (National Institutes of Health, *n*=23–25 cells per group). *P<0.05; **P<0.01 using one-way ANOVA with Bonferroni *post hoc* testing. (**b**) Representative fluoromicrographs of cells measured in (**a**). Blue is DAPI nuclear stain. Bar=10 *μ*m. Arrows indicate aggregates. (**c**) WT and KI cell lysates were prepared from WT and KI primary neurons treated with 15dPGJ2 or Vehicle (V) for 24 h and fractioned into RIPA-soluble and -insoluble fractions. (**c**) Representative immunoblots detecting poly-ubiquitin (left group) and Ubiquitin K48 (right group) in each fraction. (**d**) Immunoblot of ubiquitin K63 level in RIPA-soluble fraction. Corresponding densitometric immunoblot analysis is shown below. Data are normalized to their respective vehicle-treated groups. *β*-actin was used as a loading control. *N*=4 per group. *P<0.05; **P<0.01 *versus* WT. (**a** and **c**) Data are means±S.E.

15dPGJ2-induced protein aggregation was investigated in primary neurons from UCH-L1 C152A KI and WT mice. Primary neurons were seeded on coverslips, and treated with 2.5 *μ*M 15dPGJ2 or vehicle for 48 h before fixation, then were immunostained with anti-ubiquitin and anti-UCH-L1 antibodies. Treatment with 15dPGJ2 significantly induced Ub protein and UCH-L1 aggregation in WT primary neurons, but this effect was abrogated in UCH-L1 C152A KI cells (Figure 5b). Ubiquitin and UCH-L1 particle sizes were measured and the numbers of large and small particles per cell were counted in both WT and KI primary neurons after 15dPGJ2 or vehicle treatment. As shown in Figure 5a, the number of ubiquitin aggregates significantly increased after 15dPGJ2 treatment in WT cells but not in KI cells. Similarly, the number of large UCH-L1 particles per cell was significantly increased in WT cells when treated with 15dPGJ2 and this effect was not observed in KI cells.

Ub protein accumulation and aggregation were also investigated in both WT and KI cells after 15dPGJ2 treatment with immunoblotting. Primary neurons were incubated with vehicle or 15dPGJ2 at 2.5 or 5 *μ*M for 24 h, and the cell lysates were sub-fractionated into radioimmunoprecipitation assay (RIPA)-soluble and -insoluble fractions. PolyUb proteins were detected in each fraction using anti-polyubiquitin antibody. As shown in Figure 5c, 15dPGJ2 treatment induced polyUb-protein accumulation in the RIPA-soluble fraction; however, this effect was significantly decreased in the UCH-L1 C152A KI cells, as compared with the WT cells. Similarly, polyUb-protein aggregation detected in the RIPA insoluble fraction was induced by 15dPGJ2 in both WT and KI cells but this effect was significantly attenuated in the UCH-L1 C152A KI cells as compared with the WT cells. Polyubiquitin chains linked from Lys(K)-48 of one ubiquitin molecule to the C-terminus of another are the most common ubiquitin linkage. Lys48-linked ubiquitins are abundant *in vivo* and act as a universal signal for proteins targeted for proteasomal degradation.^16^ K48-polyUb is the most abundant ubiquitin linkage type in cells and has an important role in the UPS. Specific K48-polyubiquitin proteins were analyzed by immunoblotting in both WT and UCH-L1 C152A KI cells. Comparable to the results for polyUb protein, K48-polyUb was significantly induced by 15dPGJ2 treatment in both RIPA-soluble and -insoluble fractions and this induction was significantly reduced in UCH-L1 C152A KI cells as compared with WT cells. Taken together, the UCH-L1 C152A mutation attenuated the disruption of the UPS induced by 15dPGJ2 treatment in primary neurons. In contrast to the K48-polyUb linkage, the K63-polyUb linkage is not associated with proteasome degradation and may have a role in endosome transport and other intracellular functions. Unlike K48-polyUb, 15dPGJ2 treatment significantly decreased K63-polyUb detected by immunoblots in WT primary neurons, and this effect was significantly attenuated in UCH-L1 C152A KI cells (Figure 5d).

### Primary neurons from UCH-L1 C152A KI mice are resistant to 15dPGJ2-induced axonal injury

**Figure 6:**
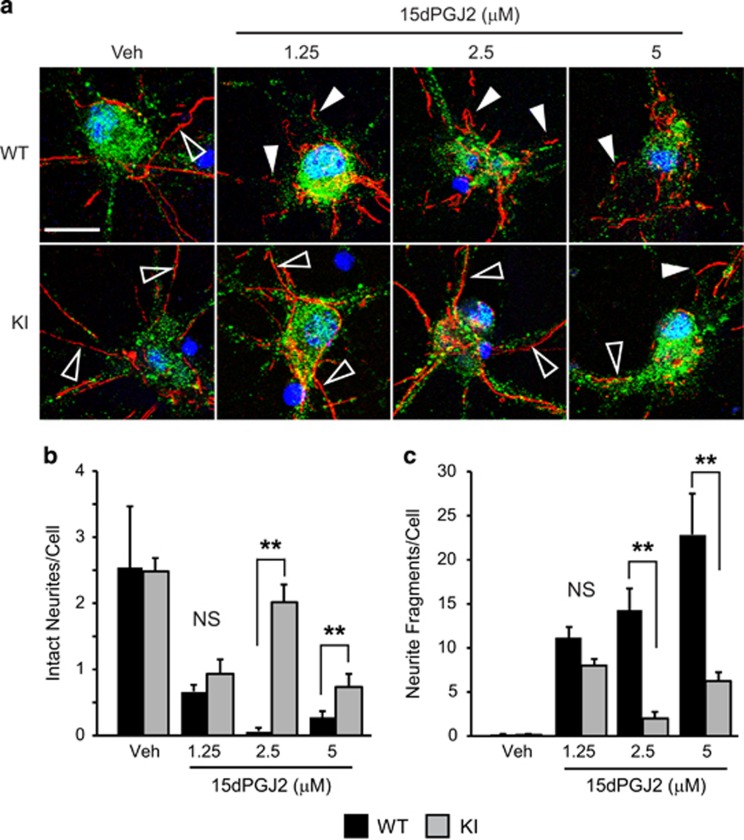
The UCH-L1 C152A mutation protects 15dPGJ2-induced injury to neurites. Wild type (WT) and UCH-L1 C152A knock-in (KI) primary neurons were incubated with 1.25–5 *μ*M 15dPGJ2 or vehicle (Veh) for 24 h then immunostained with anti-NeuN (green) and anti-neurofilament L (red) antibodies. Blue is DAPI nuclear stain. (**a**) Representative confocal fluoromicrographs taken at 240 × . Bar=20 *μ*m. (**b** and **c**) Intact neurites (outlined arrows) and neurite fragments (solid arrows) were counted in eight fields per group and are normalized to the number of cells examined. Data are means±S.E. **P<0.01 *versus* WT; NS, not significant

To determine the role of the UCH-L1 C152A mutation in 15dPGJ2-induced neuronal cell axonal injury, primary neurons from UCH-L1-C152A KI or WT mice were seeded on coverslips and incubated with 15dPGJ2 (1.25–5 *μ*M) for 24 h. The neurites were visualized by immunostaining with anti-neurofilament L antibody. As shown in Figure 6, neuronal neurites were more sensitive to lower doses of 15dPGJ2 than whole cells: neurite injury was observed when cells were treated with 1.25 *μ*M 15dPGJ2 in WT neuronal cells compared with 7.5 *μ*M 15dPGJ2 required to induce cell death. 15dPGJ2 treatment significantly decreased the number of intact neurites and increased broken fragments in WT cells in a dose-dependent manner. Neurite damage was significantly reduced in UCH-L1 C152A mutant cells, suggesting that the UCH-L1 C152A mutation preserves axonal integrity against 15dPGJ2-induced toxicity.

## Discussion

The major findings of this study are (1) CyPGs such as 15dPGJ2 adduct the C152 of UCH-L1 and mutation of the C152 of UCH-L1 attenuates the loss of hydrolase activity after 15dPGJ2 treatment; (2) the UCH-L1 C152A mutation decreases 15dPGJ2-induced accumulation and aggregation of UCH-L1 and ubiquitinated proteins in primary neurons; and (3) primary neurons derived from UCH-L1-C152A mutant mice are resistant to cell death and neurite injury induced by treatment with 15dPGJ2.

MS/MS analysis (Figure 1a) indicates that 15dPGJ2 covalently adducts the C152 cysteine of UCH-L1. This finding correlates with the observation that CyPG binding to C152 of UCH-L1 by 15dPGJ2 dramatically alters the 2D^1^H-^15^N HSQC spectrum of recombinant UCH-L1 protein consistent with unfolding of the enzyme.^15^ The effect of CyPG binding upon protein structure was abolished by mutation of C152, but not by mutation of other UCH-L1 cysteines.^15^ UCH-L1 hydrolyzes ubiquitin from poly-ubiquitinated proteins and thus may be a component of the neuronal UPS.^4, 7, 17, 18, 19^ The UCH-L1 C152A mutation tempered but did not abolish the decrease in hydrolase activity after incubation with 12.5 *μ*M 15dPGJ2. Mutation of C152A prevents the unfolding of UCH-L1 induced by CyPGs, thus preserving protein function.^15^ But the UCH-L1 hydrolase active site contains a cysteine (C90) that also maybe adducted by 15dPGJ2. Accordingly, mutation of C152 only partially prevents the loss of hydrolase activity produced by CyPGs. The C152A mutation may exert its protective effects through mechanisms in addition to hydrolase activity. UCH-L1 may have other neuron-specific functions including regulation of axon transport and synaptic function.^4, 20, 21^ Unfolding of UCH-L1 induced by CyPGs may have an important role in disrupting these functions, and these functions may be dependent upon protein–protein interactions with other components of the axon and synaptosome.^15^

CyPGs are reactive lipid species that have been shown to have a wide variety of potential protein targets.^8, 22, 23, 24^ Their reactive carbonyl group can adduct cysteine in many proteins and have been proposed to have significant biological effects in many signal transduction pathways.^25, 26, 27, 28^ CyPGs have been shown to induce cell death in neurons characterized by apoptosis and accumulation of Ub proteins.^10, 29, 30, 31^ The results in Figure 4 indicate that the C152A mutation of UCH-L1 significantly reduces 15dPGJ2 toxicity, suggesting that binding of CyPGs to this site is a significant origin of CyPG neuronal toxicity. However, the C152A UCH-L1 mutation affords incomplete protection, thus other targets of CyPGs such as binding to proteasomal proteins may contribute to their toxicity^12, 32^ The concentration of 15dPGJ2 has been measured to increase to an average concentration of 100 nM in ischemic brain,^33, 34^ but this concentration is less than what is reported to produce toxicity in culture. However, whole brain measures of CyPG concentrations may not be reflective of localized intracellular concentrations. Additionally, there are a variety of other reactive lipid species produced under pathological conditions, which also contain reactive carbonyl groups that may interact with the C152 site. Thus, binding of reactive lipids to the C152 site may be important to the pathogenesis of ischemic injury and related disorders.

Mutation of the C152 CyPG adduction site of UCH-L1, the site identified with conformational change of the protein with CyPG adduction, afforded protection from 15dPGJ2-induced cell death in primary cortical neurons. The UCH-L1 C152A mutation also attenuated the accumulation of both soluble and insoluble Ub proteins within the neurons. There are increased numbers of aggregates containing Ub proteins and UCH-L1 within the neuron after 15dPGJ2 treatment that are also attenuated by the C152A mutation. An increase in soluble Ub proteins may occur when there is either an increase in the number of misfolded proteins to be labeled for proteasomal degradation, or there is dysfunction in the UPS resulting in impaired de-ubiquitination or processing of Ub-tagged proteins by the proteasome. These data suggest that a significant portion of the effect of CyPGs upon accumulation of RIPA-soluble Ub proteins is due to its binding to UCH-L1. UCH-L1 hydrolyzes ubiquitin from polyUb-tagged proteins allowing the tagged protein to enter the proteasome and recycle free ubiquitin.^19^ Similarly, UCH-L1 has been proposed to ligate ubiquitin to proteins;^2^ thus, UCH-L1 may have a major role in the neuronal UPS. The UCH-L1 C152A mutation also attenuated the accumulation of insoluble Ub proteins and specifically K48 polyUb proteins, and decreased the number of intraneuronal particles containing polyUb proteins and UCH-L1. Accumulation of insoluble Ub proteins disrupts multiple protein degradation pathways including both the UPS and autophagy, and results in formation of large protein aggregates.^35^ Binding of CyPGs to UCH-L1 produces dramatic structural changes in the molecule and produces aggregation of the protein *in vitro.*^15^ Accumulation of insoluble protein aggregates occurs in neurodegenerative disease, such as PD, AD, and ischemic brain injury.^35, 36, 37^ Modification of UCH-L1 by CyPGs and other reactive lipids may result in aggregation of UCH-L1 and insoluble protein aggregates, and thus could be important in the pathogenesis of these and related disorders.^38^

K63-linked Ub proteins were also detected in WT and KI cells by immunoblotting (Figure 5d). Unlike K48-ubiquitin linkage, it has been suggested that K63 ubiquitin linkage does not have a significant role in the UPS, but rather is important in endosomal trafficking, DNA damage response, and intracellular signal transduction.^39, 40^ A recent report has demonstrated that impairment of K63-polyUb response during oxidative stress affects polysome stability and protein expression, rendering cells more sensitive to stress.^41^ Twenty-four hour treatment with 15dPGJ2 significantly decreased the K63-polyUb level in WT primary neurons, and this effect was significantly attenuated in UCH-L1 C152A KI cells. Therefore, besides the effects on the proteasome system, the UCH-L1 C152A mutation may protect primary neurons against CyPG insults through other mechanisms such as protection against oxidative stress.

While UCH-L1 may have a role in the neuronal UPS, there are other enzymes expressed in brain such as UCH-L3 with much higher hydrolase activity that are known to be major constituents of the UPS in other cell types.^42^ UCH-L1 is selectively expressed in neurons, suggesting that it has a neuron-specific function. UCH-L1 interacts with cytoskeletal and synaptic proteins, and thus may have an important role in axonal transport and synaptic function.^7, 43, 44, 45, 46^ Humans with mutations in UCH-L1 have been reported to have extensive white-matter abnormalities.^5^ Furthermore, UCH-L1 activity modulates LTP in hippocampus and is deficient in a mouse model of AD.^4, 47^ These data indicate that neuronal processes are more sensitive to 15dPGJ2-induced injury than the neuronal cell body and that the UCH-L1 C152A mutation protects neuronal processes from the toxic effects of CyPGs. Thus, UCH-L1 may have an important role in maintaining the structure and function of axons and dendrites.

These results demonstrate that the binding of CyPGs and other reactive lipid species may have a role in the pathogenesis of ischemia and neurodegenerative disease. Gong *et al.*^4^ have shown that it is feasible to construct a HIV-1-trans-activating (TAT) domain fused UCH-L1 protein (TAT-UCH-L1). TAT-UCH-L1 proteins cross the blood brain barrier, transduce neurons, and improve memory function in AD mouse models. Additional modification of the C152 by the TAT-UCH-L1 protein may confer additional potency and stability to these proteins enhancing their therapeutic potential. Furthermore, conventional drugs that compete for the binding of the reactive lipids to the C152 site could be designed.

In summary, CyPGs and other reactive lipids produced in cerebral ischemia may bind to UCH-L1 and disrupt its structure and function, and the metabolism of Ub proteins in the neuron. The resultant accumulation of insoluble protein aggregates may produce pathogenic effects similar to those seen in stroke and neurodegenerative diseases. Neuronal processes are particularly sensitive to CyPG toxicity mediated through CyPG binding to UCH-L1. Binding of CyPGs and other reactive lipid species to UCH-L1 may be important in the pathogenesis of stroke and neurodegenerative disease, and is a potential target for future therapeutic interventions.

## Materials and Methods

### Experimental procedures

Animal studies were performed with the approval of the University of Pittsburgh Institutional Animal Care and Use Committee and conducted according to the National Institutes of Health Guide for the Care and Use of Laboratory Animals. Animals were housed in a temperature- and humidity-controlled environment with 12 h light cycles and free access to food and water.

### Reagents and antibodies

15dPGJ2 was purchased from Cayman Chemical (Ann Arbor, MI, USA). Antibody sources were as follows: mouse monoclonal anti-mono- and poly-ubiquitinated proteins antibody (clone FK2) and anti-poly-ubiquitinated proteins antibody (clone FK1) were from Enzo Life Sciences (Plymouth Meeting, PA, USA). Monoclonal anti-ubiquitin antibody was from Covance (Berkeley, CA, USA) and anti-ubiquitin Lys48-specific antibody was from Millipore (Temecula, CA, USA), anti-ubiquitin Lys63-specific antibody was from Abcam (Cambridge, MA, USA); anti-PARP, anti-caspase-3, and anti-Neurofilament-L antibodies were from Cell Signaling (Boston, MA, USA); anti-*β*-actin antibody and anti-UCH-L1 antibody were from Sigma-Aldrich (St Louis, MO, USA); anti-NeuN antibody was from Millipore; and anti-GAPDH antibody was from Ambion (Grand Island, NY, USA). Cy3- and Alexafluor 488-conjugated secondary antibodies were from Jackson Immunoresearch Lab (West Grove, PA, USA). Ultra performance liquid chromatography organic solvents and water were from VWR (West Chester, PA, USA). The lentiviral expression vector, pLVX-IRES-ZsGreen1 vector, and Lenti-X HTX concentrator and packaging system were purchased from Clontech Laboratories (Mountain View, CA, USA). WST-1 cell proliferation assay kit and Lenti-X 293 T cell line were purchased from Clontech.

### Plasmid constructs

The DNA sequence encoding full-length rat UCH-L1 WT was amplified by PCR, and cloned into pET22b vector (Novagen, San Diego, CA, USA), using NdeI and *Xho*I restriction sites. A UCH-L1 point mutation substituting alanine for cysteine (UCH-L1 C152A) was introduced by PCR. Constructs were confirmed by sequencing. For lentiviral expression vector construction, UCH-L1 WT or UCH-L1 C152A cDNA was inserted into pLVX-IRES-ZsGreen1 vector with *Xho*I and *Bam*HI restrictive sites to generate lenti-UCH-L1 WT or lenti-UCH-L1 C152A.^24^ This vector expresses UCH-L1 WT or its mutant C152A and the green fluorescent protein ZsGreen from a bicistronic mRNA transcript, allowing ZsGreen to be used as an indicator of transduction. The resulting constructs were confirmed by DNA sequencing. To generate infectious lentiviral particles, Lenti-X 293 T cells were transfected with the lenti-UCH-L1 WT and KI vectors, or empty lentiviral vector together with Lenti-X packaging mix using XFect (Clontech) following the manufacturer's instructions. Lentiviral particles were then collected, concentrated, and purified. UCH-L1 C152A and WT lentiviral titrations were determined with a Lenti-X qRT-PCR titration kit (Clontech).

### Expression and purification of recombinant proteins

Plasmids were introduced into *Escherichia coli* Rosetta (DE3) strains (Novagen) producing 6 × His-tagged full-length UCH-L1 WT or the mutant protein UCH-L1 C152A. Protein production was induced by shaking the *E. coli* Rosetta cells in ‘overnight express autoinduction system' (Novagen) at 300 r.p.m. for 16 h, then purified with a His-select column (Sigma-Aldrich) according to the manufacturer's protocol. Protein purity was assessed by SDS-PAGE and Coomassie blue staining. Recombinant UCH-L1 WT and UCH-L1 C152A proteins were further confirmed by immunoblotting.

### Protein in-gel digestion and tandem MS

Rat primary neurons were infected with LV-Flag UCHL-WT at DIV2 to overexpress Flag-tagged UCH-L1-WT. Cells were then incubated with 15dPGJ2 (20 *μ*M) for 24 h at DIV11 before harvest. The cell lysates were immunoprecipitated with an anti-Flag antibody, and the resulting precipitants were subjected to polyacrylamide gel electrophoretic separation, in-gel digestion, and nanoLC-MS and -MS/MS analyses.

The gel was then stained with Coomassie blue, protein bands were excised, minced, and digested with porcine Trypsin Gold (Promega, Madison, WI, USA), as previously described^24, 48^ with minor modifications. Peptides extracted from the digested gel bands were analyzed on a Thermo Fisher LTQ Orbitrap Velos (Thermo Fisher, Pittsburgh, PA, USA) connected to a Waters Acquity Ultra Performance Liquid Chromatography (UPLC) system (Waters Corp., Milford, MA, USA) consisting of sample trap (nanoAcuity UPLC trap column, 180 *μ*m × 20 mm, 5 *μ*m particle size, Symmetry C18, Waters Corp.) and a C18 column (nanoAcquity UPLC column, 75 *μ*m × 250 mm, 1.7 *μ*m particle size, BEH300 C18, Waters Corp.) interfaced with a nanospray ionization source. Solvent A was 0.1% formic acid in water. Solvent B was 0.1% formic acid in acetonitrile. Samples were loaded on the sample trap with 1% Solvent B at a flow rate of 15 *μ*l/min for 1 min. Peptides were then eluted off the column with a 90-min gradient running at 300 nl/min: 5% B for 3 min, 5–55% B in 60 min, 30–95% B in 1 min, 95% B for 5 min, 95–5% B in 1 min, 5% B for 20 min. All MS spectra were acquired in the Orbitrap detector at a resolution of 60 000 in profile mode, while top nine data-dependent MS/MS spectra were yielded by fragmentation of precursor peptide ions using collision-induced dissociation (CID) normalized collision energy setting of 35% in the ion trap of the LTQ mass spectrometer, then acquired in the Orbitrap at a resolution of 7500.

Database searches were done with Proteome Discoverer 1.4 against the complete Uniprot human database appended to a contaminant database (88 575 sequences, 35 106 826 residues) with the Sequest search engine for a trypsin digest with two missed cleavages, three dynamic modifications (oxidation of methionines, carbamidomethylation of cysteines, and 15dPGJ2 adduction of cysteines). The mass tolerance was set at 10 p.p.m. for precursor mass and 0.8 Da for CID fragment ion masses. Peptide identifications were validated using the Percolator algorithm with a 5% global false discovery rate (FDR). The spectra of 15dPGJ2-adducted peptides were manually inspected.

### *In vitro* hydrolase activity assay

The UCH-L1 hydrolase activity assay was performed as previously described with slight modifications.^14^ Briefly, 2 *μ*M UCH-L1 protein was incubated with 12.5 *μ*M 15dPGJ2 or vehicle for 2 h before dilution with UCH-L1 hydrolase buffer. Samples were then mixed with 500 nM ubiquitin-AMC (BostonBiochem, Cambridge, MA, USA). The final concentration for UCH-L1 was 100 nM. Free AMC fluorophore was read using a fluorescent plate reader (ex 360 nm, em 460 nm).

### Primary neuronal cell culture and LV infection

Rat cortical primary neuronal cultures were prepared from E17 fetal rats (Sprague-Dawley, Charles River, Wilmington, MA, USA) as previously described^49, 50^and used for experiments after 9 DIV. Cells were grown in serum-free Neurobasal medium (Invitrogen, Carlsbad, CA, USA) supplemented with B27 and GlutaMAX (Invitrogen). To overexpress UCH-L1 or mutant UCH-L1 C152A in primary neurons, cells were seeded in 96-well plates and lenti-UCH-L1, lenti-UCH-L1 C152A, or empty lentiviral particles were added to the culture medium at 1.2 × 10^8^ particles/well for 18 h. Viral infection was performed at DIV 2, and cells underwent 15dPGJ2 treatment at DIV10. Cell viability and cell death were quantitatively assessed by LDH assay and WST-1 cell viability assay according to the manufacturer's protocols (*n*=6–12 wells per group).

### Construction of the UCH-L1 C152A KI mouse

In collaboration with the University of Michigan Transgenic Core, the BAC technique was used to construct a UCH-L1 C152A KI mouse.^51, 52, 53, 54^ Homologous recombination of DNA fragments was used to modify a BAC containing UCH-L1 to produce a point mutation in UCH-L1 that converts the 152 cysteine to alanine (TGT>GCT) within the normal chromosome of the mouse (Figure 3a). In brief, a C57BL/6J mouse genomic clone from the RP23 BAC library was modified to carry the TGT>GCT mutation and a drug selection cassette (PGK-neo) flanked by flippase recognition target (FRT) sites. The drug selection cassette confers resistance to the antibiotic G418. The FLP recombinase enzyme recognizes FRT sites and is used to remove the cassette so that it will not interfere with UCH-L1 C152A gene expression. Thus, the UCH-L1 C152A mutation is transcribed under control of the original physiological promoters and other flanking sequences of the mouse chromosome to faithfully reproduce physiological expression of mutant UCH-L1 C152A. The targeting vector was constructed by gap repair from the BAC and was confirmed by sequencing: (1) the TGT>GCT mutation was correctly positioned in exon 6; (2) the PGK-neo cassette was correctly inserted; (3) both FRT sites were present around the PGK-neo cassette, and the diagnostic *Nsi* I restriction enzyme site was present on the 3′ side of the 3′ FRT site, permitting the use of *Nsi* I for the identification of gene-targeted embryonic stem (ES) cells. The vector was then linearized and electroporated into mouse C57BL/6J ES cells. After neomycin selection, Southern blots were performed to identify ES cells having homologous recombination. These ES cells were shipped to Taconic Artemis (Koeln, Germany) and injected into blastocysts to generate heterozygous UCH-L1 C152A KI mice on a C57Bl6 background. Male heterozygous UCH-L1 C152A mice were then bred with female C57Bl6 mice. Heterozygous offspring were crossed to produce homozygous UCH-L1 C152A and WT UCH-L1 lines. Mouse genotype was identified by genomic DNA extraction and PCR amplification with primers 5′-CGAGGTAATAGAAAATGCTTCTGG-3′ and 5′-GAATATGCTCCAAACAGCTTGC-3′. The details of targeting vector, Southern blotting, and genotyping protocols are available upon request. Mouse cortical primary neuronal cultures were prepared from E17 WT and KI fetal mice as described above and used for experiments after 9 DIV.

### Fluorescent immunocytochemistry

Primary neurons were seeded on glass coverslips and incubated with 15dPGJ2 or methyl acetate (veh) for 24 h before fixation with 4% PFA in phosphate-buffered saline (PBS). Immunocytochemistry using anti-Ub-proteins and anti-UCH-L1 antibodies followed by Alexafluor 488 and DyLight 549-conjugated secondary antibodies was performed, and cells were photographed using an Olympus confocal microscope with FluoView FV1000 software (Olympus, Tokyo, Japan). For particle size counting, cells were photographed at × 240. UCH-L1 particles <0.5 *μ*M^2^ and ≥0.5 *μ*M^2^ were counted in 23–25 individual cells per group using NIH ImageJ software (National Institutes of Health, Bethesda, MD, USA). Data were analyzed by a blinded investigator using one-way ANOVA with Bonferroni *post hoc* testing.

### Preparation of detergent-insoluble fraction from cell lysates

The cell lysate detergent-insoluble protein aggregate fraction was prepared as previously described.^34^ In brief, primary neurons were treated with vehicle (methyl acetate) or 15dPGJ2 (2.5 or 5 *μ*M) for 24 h. Cells were lysed in RIPA lysis and extraction buffer (Pierce, Rockford, IL, USA) containing 25 mM Tris-HCl (pH 7.6), 150 mM NaCl, 1% NP-40, 1% sodium deoxycholate, 0.1% SDS, and protease inhibitor cocktail (Pierce). Cells were incubated with RIPA buffer for 15 min on ice then centrifuged at 16,800 × *g* at 4 °C for 15 min. The supernatants were collected and the insoluble pellets were washed twice with cold PBS, and then re-suspended in RIPA buffer containing 0.5% SDS for 1 h on ice and sonicated three times for protein solubilization. Protein concentrations were determined by the bicinchoninic acid (BCA) method (Pierce).

### Western blotting

Western blotting was performed as previously described.^14^ For caspase-3, PARP, and UCH-L1 detection, cell lysates were resolved on 10 or 12% SDS-PAGE. After blocking with 5% non-fat milk in TBS/Tween-20, membranes were incubated with anti-caspase-3, anti-PARP, or anti-UCH-L1 antibodies at 4 °C overnight. For Ub-protein detection, cell lysates were resolved on a 4–20% linear gradient polyacrylamide gel (Bio-Rad, Hercules, CA, USA) before incubation with anti-poly-ubiquitinated conjugates, anti-ubiquitin Lys48-specific or anti-ubiquitin Lys63-specific antibodies (1:1000 for all). Blots were washed and the appropriate secondary antibodies applied. Protein signal was visualized with ECL reagents (Pierce). Blots were subsequently stripped and re-probed using anti-GAPDH or *β*-actin antibodies for verification of equal protein loading. Brain cortex, hippocampus, and striatum were dissected from male 12-week-old WT and KI mice (*n*=4 per group) and rapidly frozen on dry ice until homogenization with T-PER tissue protein extraction reagent (Pierce) and protein measurement. Equal amounts of protein were loaded for SDS-PAGE and immunoblotted using anti-UCH-L1 antibody as described above.

### Axonal injury assay

Primary neurons from WT or UCH-L1 C152A KI mice were seeded onto l-poly d-lysine-coated glass coverslips. 15dPGJ2 treatment was performed on DIV11, and cells were fixed with 4% PFA in PBS at different time points (8, 16, and 24 h post treatment). After permeabilization with blocking buffer (2% BSA, 1% fish skin gelatin and 0.02% saponin in PBS) for 1 h at room temperature, cells were immunostained with anti-Neurofilament-L antibody (1 : 100, Cell Signaling Technology #2837, Danvers, MA, USA) and Alexafluor 488-conjugated secondary antibody sequentially and photographed using a confocal microscope (Olympus, Tokyo, Japan). Image acquisition and analysis was performed on × 60 images randomly taken from two coverslips for each treatment condition per genotype (*n*=8 fields per group). Quantification of axon degeneration was performed by a blinded investigator as previously described.^55, 56^ The axon/neurite outgrowth was evaluated based on morphological characteristics: (1) intact: length of neurite outgrowth >2 × the length of the soma and (2) fragmented: isolated neurite swellings. The numbers of intact neurites and neurites fragments were normalized to the number of neurons examined.

### Statistical analysis

Data are expressed as means±S.E. and were analyzed using one-way ANOVA with Bonferroni or Dunnett's *post hoc* testing where appropriate using SPSS software (IBM Corporation, Armonk, NY, USA). Results were considered to be significant when *P*<0.05.

## Abbreviations

15dPGJ2: 15-deoxy-Δ^12,14^-prostaglandin J_2_
AD: Alzheimer's disease
BAC: bacterial artificial chromosome
CyPG: cyclopentenone prostaglandin
DIV: days *in vitro*
EGFP: enhanced green fluorescence protein
FRT: flippase recognition targets
GAPDH: glyceraldehyde 3-phosphate dehydrogenase
KI: knock-in
LDH: lactate dehydrogenase
LV: lentivirus
MS: mass spectrometry
NS: not significant
PAGE: polyacrylamide gel electrophoresis
PARP: poly ADP ribose polymerase
PBS: phosphate-buffered saline
PD: Parkinson's disease
PFA: paraformaldehyde
RIPA: radioimmunoprecipitation assay
SDS: sodium dodecyl sulfate
SE: standard error
TAT: HIV-1 Trans-activator of transcription
Ub: ubiquitinated
UCH-L1: ubiquitin-C-terminal hydrolase
UPS: ubiquitin-proteasome system
V: vehicle
WST-1: cell proliferation reagent
WT: wild type
